# Impact of diagnosis-related group–based bundled payment on hospitalization costs and clinical outcomes in total hip arthroplasty: an interrupted time series analysis from Wenzhou, China

**DOI:** 10.3389/fpubh.2026.1886645

**Published:** 2026-08-20

**Authors:** KeCheng Li, XiaoChan Xiang, Weiwei Zeng, Ge Li, JianWu Shi, XiaoLi Gao, QianWen Ye

**Affiliations:** Quality Control Office, Ruian People's Hospital, Wenzhou, Zhejiang, China

**Keywords:** bundled payment, China, cost containment, diagnosis-related groups, health payment reform, interrupted time series, orthopedic implant procurement, total hip arthroplasty

## Abstract

**Background:**

China's nationwide rollout of diagnosis-related group (DRG) bundled payment, implemented from January 2021, is one of the most consequential structural reforms to hospital reimbursement in recent decades. Total hip arthroplasty (THA)—a high-volume, implant-intensive elective procedure with marked inter-institutional cost variation—provides an analytically tractable context in which to evaluate whether fixed-payment incentives alter provider resource utilization while preserving clinical quality.

**Methods:**

This retrospective longitudinal study included 2,621 consecutive THA admissions at a tertiary teaching hospital in Wenzhou, Zhejiang Province, between January 2015 and April 2026 (pre-DRG: *n* = 1,041; post-DRG: *n* = 1,580). Segmented linear regression with Newey–West heteroscedasticity- and autocorrelation-consistent (HAC) standard errors was applied to 136 monthly aggregated observations (72 pre-intervention; 64 post-intervention). Primary outcomes were monthly mean total hospitalization cost, drug cost, and orthopedic implantable device cost. A composite adverse discharge outcome rate (in-hospital mortality, failure to achieve clinical improvement, and unclassified discharge) was the clinical quality endpoint. Four pre-specified sensitivity analyses addressed consumer price index (CPI) inflation adjustment, COVID-19–related confounding (exclusion of 2020), Prais–Winsten first-order autoregressive [AR(1)] correction, and case-mix adjustment for monthly patient age and sex composition.

**Results:**

Mean total hospitalization cost declined from CNY 53,476 ± 14,898 to CNY 37,475 ± 15,273 (−29.9%; *p* < 0.001). Orthopedic implantable device cost fell from CNY 35,826 ± 9,607 to CNY 17,727 ± 10,097 (−50.5%; *p* < 0.001), and drug cost from CNY 6,685 ± 5,058 to CNY 5,741 ± 3,311 (−14.1%; *p* < 0.001). Interrupted time series (ITS) modeling identified strong pre-existing downward trends in both total (β_1_ = −89.79 CNY/month; *p* = 0.003) and device costs (β_1_ = −104.81 CNY/month; *p* = 0.001) prior to DRG implementation. Post-DRG slope changes for total and device costs did not reach statistical significance (*p* = 0.071 and *p* = 0.061, respectively). Drug cost exhibited a significant immediate level increase (β_2_ = 3,006 CNY; *p* = 0.003) followed by a statistically significant sustained monthly decline (β_3_ = −27.80 CNY/month; *p* = 0.045). The composite adverse discharge rate did not differ significantly between periods (1.06% vs. 0.63%; χ^2^ = 0.944; *p* = 0.334). Sensitivity analysis excluding 2020 yielded a statistically significant post-DRG slope change for total cost (β_3_ = −146.92 CNY/month; *p* = 0.047), suggesting that COVID-19–related disruptions partially attenuated this effect in the primary analysis.

**Conclusions:**

Following DRG implementation, total and orthopedic implantable device costs continued their pre-existing downward trajectories, with observed expenditure sustained well below the pre-intervention counterfactual. The primary ITS analysis did not identify a statistically significant DRG-specific acceleration of this decline for total or device costs, a finding that predominantly reflects the dominant contribution of antecedent volume-based procurement reform. Drug cost exhibited the most clearly DRG-attributable effect—an initial transitional surge followed by sustained statistically significant decline—compatible with institutional adaptation after implementation of fixed-payment incentives. No evidence of in-hospital deterioration in adverse discharge outcomes was detected; this underpowered composite endpoint did not capture post-discharge events such as 30-day readmission or surgical-site infection.

## 1 Introduction

Musculoskeletal disorders collectively represent one of the leading causes of global disability, with age-standardized disability-adjusted life-years increasing by 14.5% between 1990 and 2019 (1). Within this burden, hip osteoarthritis and femoral neck fracture carry particular epidemiological and economic weight: both conditions are rising in prevalence as populations age, and both have total hip arthroplasty (THA) as the definitive surgical intervention. Globally, THA volumes continue to grow at 5%−10% annually, and in China alone the procedure approaches one million cases per year, generating substantial direct hospitalization expenditure dominated by prosthetic implant costs—which may account for 60%−70% of the total case cost in Chinese tertiary centers (2, 3).

Within China, THA costs vary markedly by clinical indication, comorbidity profile, and institutional characteristics (3), creating an environment in which payment system design exerts outsized influence on resource allocation. For decades, Chinese public hospitals operated under fee-for-service (FFS) reimbursement—an architecture that incentivises over-provision of diagnostic tests, pharmaceuticals, and high-value implants, and has been associated with unsustainable cost escalation, misaligned provider incentives, and documented pharmaceutical over-prescribing (4, 5). The systemic consequences of FFS are well-documented: by creating marginal revenue from each additional unit of care delivered, FFS structurally rewards volume expansion rather than value creation.

To address these distortions, the National Healthcare Security Administration (NHSA) mandated nationwide adoption of diagnosis-related group (DRG) bundled payment from January 2021 (6). Under DRG reimbursement, hospitals receive a fixed case-rate payment calibrated to nationally derived tariffs; any expenditure exceeding the tariff is borne by the institution, thereby transferring financial risk to providers and creating institutional incentives for care standardization and resource stewardship. Concurrently, China's national volume-based procurement (VBP) programme for orthopedic implants achieved centrally tendered price reductions of 50%−80% for hip prosthetic components between 2019 and 2021 (3, 7)—a supply-side shock that intersects temporally with DRG adoption and must be explicitly modeled in any rigorous policy evaluation.

Whether DRG reform simultaneously achieves cost containment and quality preservation is empirically contested. A systematic review of DRG effects in Chinese hospitals found modest efficiency gains but identified concerns about diagnostic up-coding, selective patient admission, and potential quality compromise for complex cases (8). Qualitative research has documented ethical dilemmas experienced by Chinese clinicians confronting fixed-payment constraints, including pressures toward premature discharge and selective case acceptance. International evidence from South Korea, England, and Australia shows heterogeneous outcomes: DRG implementation has reduced length of stay across settings but has also altered case-mix composition and, in under-resourced payer environments, has incentivised risk-averse clinical behaviors (9, 10).

Despite this growing evidence base, a critical methodological gap persists: most evaluations rely on simple before–after comparisons that cannot distinguish DRG-specific effects from contemporaneous secular trends or co-interventions. In the Chinese THA setting, the temporal coincidence of DRG adoption with VBP makes attribution especially challenging: any design that does not explicitly model pre-existing trends risks conflating procurement-driven cost reductions with DRG-specific behavioral change. Interrupted time series (ITS) analysis provides the strongest feasible quasi-experimental design for this purpose, partitioning the observed trajectory into pre-existing trend (β_1_), immediate level change at the policy date (β_2_), and post-intervention slope change (β_3_) (11, 12).

No published study has applied ITS analysis to THA costs and clinical outcomes using a continuous 11-year time series in a Chinese institutional context. The present study addresses this gap using a deduplicated cohort of 2,621 THA admissions from a tertiary teaching hospital in Wenzhou between January 2015 and April 2026. Our primary objectives were: (i) to estimate DRG-associated ITS effects on total hospitalization cost, drug cost, and orthopedic implantable device cost; and (ii) to assess whether cost changes were accompanied by deterioration in adverse discharge outcomes. Secondary objectives included characterizing the drug cost trajectory under fixed-payment incentives and evaluating robustness across four pre-specified sensitivity analyses.

## 2 Methods

### 2.1 Study setting, data source, and ethics

**Figure 1:**
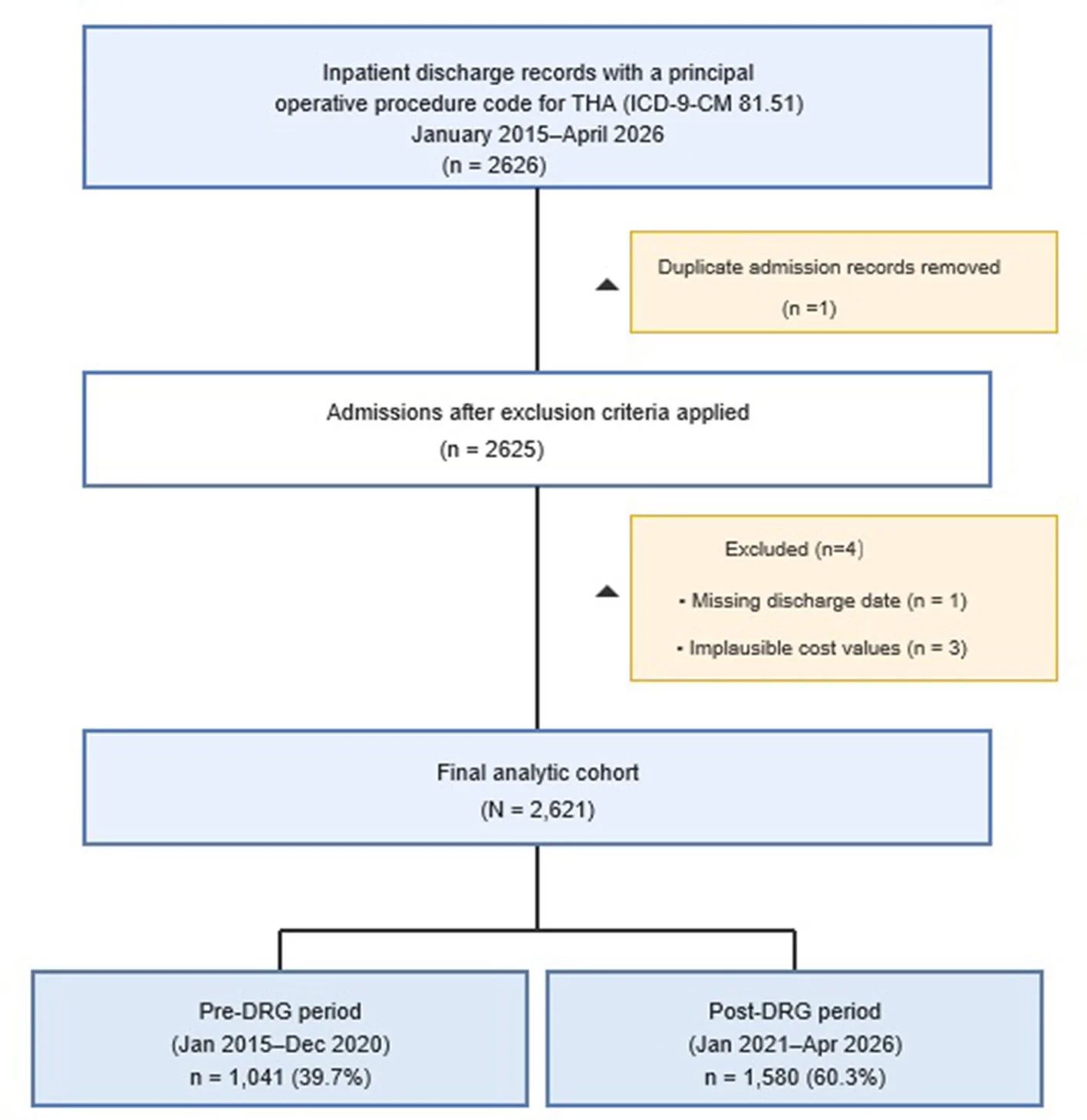
Patient flow diagram. Of the initial 2,626 records retrieved, 1 duplicate entry and 4 records with missing discharge dates or implausible cost values were excluded, yielding 2,621 unique THA admissions for analysis.

This retrospective longitudinal study was conducted at Ruian People's Hospital, a tertiary teaching hospital affiliated with Wenzhou Medical University and a high-volume regional arthroplasty center. Administrative discharge records for all inpatient THA admissions between January 2015 and April 2026 were extracted from the hospital information system. Inclusion required a principal operative procedure code for THA (ICD-9-CM 81.51). Records with missing discharge dates or implausible cost values were excluded, yielding a final analytic cohort of 2,621 admissions (Figure 1). Of the initial 2,626 inpatient discharge records with a principal operative procedure code for THA (ICD-9-CM 81.51), 1 duplicate admission record was removed and 4 records were excluded because of missing discharge date (*n* = 1) or implausible cost values (*n* = 3), yielding a final analytic cohort of 2,621 admissions (exclusion rate 0.19%). The excluded records were too few to permit formal statistical comparison with the analytic cohort. Given the extremely low exclusion rate and the administrative nature of the missing fields, the data are most consistent with Missing Completely at Random (MCAR). No systematic difference in exclusion frequency was observed before vs. after DRG implementation. No individual patient identifiers were retained.

Ethical approval was obtained from the Institutional Review Board (IRB) of Ruian People's Hospital (Approval No. M2025002) prior to data extraction and analysis. The study used previously collected, de-identified clinical data generated during routine care. The IRB waived the requirement for individual informed consent due to the retrospective design and use of anonymized data, as no research-specific interventions were performed. All procedures complied with the Declaration of Helsinki and institutional guidelines for secondary use of clinical records.

### 2.2 Policy intervention

The intervention was DRG-based bundled payment for THA admissions, adopted in January 2021 in accordance with the NHSA national mandate (6). The intervention date was externally determined by regulatory policy and independent of hospital-level outcomes, satisfying a key identifying assumption of ITS analysis. The concurrent VBP programme for orthopedic implants, progressively implemented between 2019 and 2021, generated independent downward pressure on implant unit prices and is a critical co-intervention explicitly accounted for in the ITS model (3, 7, 13, 14).

### 2.3 Outcome variables

Monthly mean values were constructed for three cost outcomes: (i) total hospitalization cost (CNY), encompassing all inpatient charges; (ii) drug cost (CNY), representing all pharmaceutical expenditure; and (iii) orthopedic implantable device cost (CNY), comprising prosthetic components, fixation hardware, and surgical consumables. All costs are in nominal CNY in the primary analysis; a CPI-adjusted sensitivity analysis deflates values to constant 2015 CNY using annual national CPI data.

The adverse discharge outcome was defined as the monthly proportion of admissions with a recorded discharge status corresponding to in-hospital mortality, failure to achieve clinical improvement, or unclassified discharge. This composite approximates early clinical failure within the available administrative data. The “unclassified discharge” component corresponds to the residual “other” disposition code recorded in the hospital information system, applied when a discharge could not be classified as cured, improved, not cured, or death (e.g., discharge against medical advice, inter-facility transfer, or an otherwise unspecified administrative disposition). It accounted for 16 of 2,621 admissions (0.6%) overall, distributed similarly across the pre-DRG (8/1,041, 0.8%) and post-DRG (8/1,580, 0.5%) periods.

### 2.4 Interrupted time series design

ITS analysis used segmented linear regression applied to 136 monthly aggregate observations, following Lopez Bernal et al. (11):


$$\begin{array}{l} Y_{t}\;=\;\beta_{0}\;+\;\beta_{1}\cdot t\;+\;\beta_{2}{Post}_{t}\;+\;\beta_{3}\dot{\left(t\times Post_{t}\right)}\;+\varepsilon_{t} \end{array}$$

**Figure 2:**
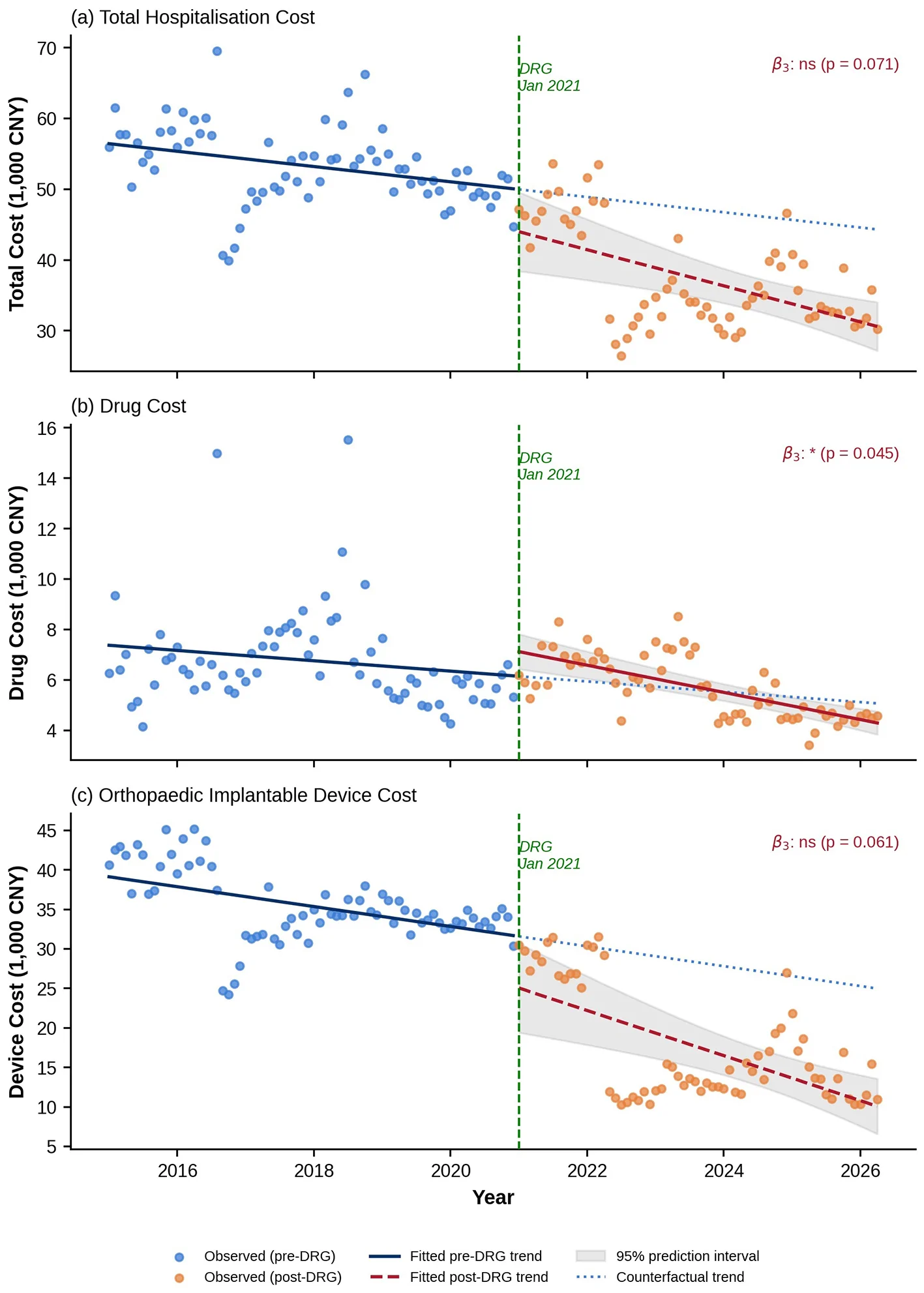
Interrupted time series analysis of monthly mean hospitalization costs, January 2015–April 2026. **(a)** Total hospitalization cost; **(b)** Drug cost; **(c)** Orthopedic implantable device cost. Circles: monthly observed means (blue, pre-DRG; orange, post-DRG). Pre-DRG fitted lines: solid. Post-DRG fitted lines: solid when slope change (β_3_) reached statistical significance at α = 0.05 (**b**, drug cost, *p* = 0.045); dashed when it did not (**a, c**, total cost *p* = 0.071; device cost *p* = 0.061). Dotted blue line: counterfactual projection of the pre-DRG trend into the post-DRG period. Light gray shading: 95% prediction interval. Vertical dashed green line: DRG implementation (January 2021). All coefficients and exact *P*-values as reported in Table 2.

Where *Y*_0_ is the mean monthly outcome at time t (*t* = 1,…,136); Post_*t*_ equals 1 from January 2021 onwards. β_0_, series baseline; β_1_, pre-intervention monthly trend; β_2_, immediate level change; β_3_, post-intervention slope change. Newey–West HAC standard errors with lag = [4(n/100)^(2/9)^] = 4 (for *n* = 136) addressed residual autocorrelation (15). A counterfactual trajectory was constructed by projecting the pre-intervention fitted line into the post-intervention period. In Figure 2, post-DRG fitted lines are drawn as dashed when β_3_ did not reach statistical significance (total and device costs) and as solid when it did (drug cost), to visually distinguish supported from non-significant slope changes. All analyses were performed in Python 3.12 (statsmodels 0.14; scipy 1.13).

### 2.5 Sensitivity analyses

Four pre-specified sensitivity analyses were conducted: (i) CPI deflation to constant 2015 CNY; (ii) exclusion of 2020 to account for COVID-19-related disruption to elective surgical activity; (iii) Prais-Winsten GLS with AR(1) error correction, given Durbin-Watson (DW) statistics below 1.5 in the primary models for total and device costs; and (iv) a case-mix-adjusted model that added monthly mean patient age and monthly proportion of female patients as covariates to the segmented regression, to test whether the significant post-DRG demographic shift (Section 3.1) confounds the primary ITS estimates.

### 2.6 Statistical analysis

Patient-level characteristics were compared using two-sample *t*-tests (continuous variables) and chi-square tests (categorical variables). Standard deviations reflect patient-level dispersion. A secondary demographic-adjusted ITS model was performed by extending the primary segmented regression with monthly mean age and the proportion of female patients as time-varying covariates. Two-sided α = 0.05 was applied throughout; 0.05 < *p* ≤ 0.10 is described as not reaching statistical significance, without “borderline” qualification in conclusions. ITS coefficients are annotated: ^***^p < 0.001, ^**^p < 0.01, ^*^p < 0.05; ns denotes *p* ≥ 0.05.

## 3 Results

### 3.1 Patient characteristics

After removal of one duplicate record and exclusion of four records with missing discharge dates or implausible cost values, 2,621 unique THA admissions remained for analysis. Of these, 1,041 admissions (39.7%) occurred during the pre-DRG period (January 2015–December 2020) and 1,580 (60.3%) during the post-DRG period (January 2021–April 2026). Patient characteristics and unadjusted cost outcomes are summarized in Table 1.

**Table 1:** Patient characteristics and unadjusted cost outcomes by DRG policy period.

Variable	Pre-DRG (Jan 2015–Dec 2020)	Post-DRG (Jan 2021–Apr 2026)	*P*-value	Total (*N* = 2,621)
Sample size, *n*	1,041	1,580	—	2,621
Age (years), mean ± SD	65.4 ± 10.4	68.9 ± 10.6	< 0.001	67.5 ± 10.7
Female sex, *n* (%)	560 (53.8%)	1,028 (65.1%)	<0.001	1,588 (60.6%)
Total hospitalization cost (CNY), mean ± SD	53,476 ± 14,898	37,475 ± 15,273	<0.001	43,831 ± 17,030
Drug cost (CNY), mean ± SD	6,685 ± 5,058	5,741 ± 3,311	<0.001	6,116 ± 4,120
Orthopedic implantable device cost (CNY), mean ± SD	35,826 ± 9,607	17,727 ± 10,097	<0.001	24,916 ± 13,287
Adverse discharge outcome, ^a^= *n* (%)	11 (1.06%)	10 (0.63%)	0.334	21 (0.80%)

^a^Adverse discharge outcome: composite of in-hospital mortality, failure to achieve clinical improvement, and unclassified discharge. P-values: two-sample t-test (continuous); chi-square test (categorical). CNY, Chinese yuan renminbi; SD, standard deviation.

Post-DRG patients were significantly older (68.9 ± 10.6 vs. 65.4 ± 10.4 years; *p* < 0.001) and included a significantly higher proportion of female patients (65.1 vs. 53.8%; *p* < 0.001).

Unadjusted mean total hospitalization cost declined from CNY 53,476 ± 14,898 to CNY 37,475 ± 15,273 (−29.9%; *p* < 0.001). Orthopedic implantable device cost fell from CNY 35,826 ± 9,607 to CNY 17,727 ± 10,097 (−50.5%; *p* < 0.001), and drug cost from CNY 6,685 ± 5,058 to CNY 5,741 ± 3,311 (−14.1%; *p* < 0.001). The composite adverse discharge rate was 1.06% pre-DRG and 0.63% post-DRG (χ^2^ = 0.944; *p* = 0.334).

### 3.2 ITS analysis of hospitalization costs

Full ITS regression results are presented in Table 2, and the segmented regression trajectories with counterfactual projections are illustrated in Figure 2.

**Table 2:** Interrupted time series regression results (Newey–West HAC standard errors).

Outcome	Parameter	Coefficient	95% CI	*P*–value
Total hospitalization cost	Intercept (β_0_)	56,522	53,142, 59,903	< 0.001
	Pre-trend (β_1_, CNY/month)	−89.79	−148.6, −31.0	0.003
	Level change (β_2_, CNY)	2,983	−11,450, 17,415	0.685
	Slope change (β_3_, CNY/month)	−122.76	−256.1, 10.5	0.071
*R*^2^ = 0.703 | DW = 0.818				
Drug cost	Intercept (β_0_)	7,393	6,419, 8,367	< 0.001
	Pre-trend (β_1_, CNY/month)	−17.07	−39.4, 5.2	0.133
	Level change (β_2_, CNY)	3,006	1,009, 5,003	0.003
	Slope change (β_3_, CNY/month)	−27.80	−54.9, −0.7	0.045
*R*^2^ = 0.225 | DW = 1.290				
Orthopedic implantable device cost	Intercept (β_0_)	39,235	35,469, 43,001	< 0.001
	Pre-trend (β_1_, CNY/month)	−104.81	−168.9, −40.7	0.001
	Level change (β_2_, CNY)	3,168	−11,601, 17,937	0.674
	Slope change (β_3_, CNY/month)	−132.93	−271.7, 5.9	0.061
*R*^2^ = 0.796 | DW = 0.436				

β_0_, estimated baseline at t = 1 (January 2015); β_1_, pre-intervention monthly trend; β_2_, immediate level change at DRG implementation (January 2021); β_3_, post-intervention slope change. HAC, heteroscedasticity- and autocorrelation-consistent; DW, Durbin–Watson statistic.

For total hospitalization cost, a statistically significant pre-existing declining secular trend was identified (β_1_ = −89.79 CNY/month, 95% CI [−148.6, −31.0]; *p* = 0.003), equivalent to approximately CNY 1,077 per year before DRG implementation. At the DRG implementation date, no statistically significant immediate level change was observed (β_2_ = 2,983 CNY; *p* = 0.685). The post-DRG slope change did not reach statistical significance (β_3_ = −122.76 CNY/month; 95% CI [−256.1, 10.5]; *p* = 0.071). The counterfactual projection in Figure 2a illustrates that observed post-DRG costs remained well below the trajectory expected under continuation of the pre-DRG trend.

For drug cost, no significant pre-DRG trend was identified (β_1_ = −17.07 CNY/month; *p* = 0.133). DRG implementation was associated with a significant immediate level increase (β_2_ = 3,006 CNY; 95% CI [1,009, 5,003]; *p* = 0.003), followed by a statistically significant sustained monthly decline (β_3_ = −27.80 CNY/month; 95% CI [−54.9, −0.7]; *p* = 0.045). For orthopedic implantable device cost, a highly significant pre-DRG declining trend dominated model fit (β_1_ = −104.81 CNY/month; 95% CI [−168.9, −40.7]; *p* = 0.001; *R*^2^ = 0.796). Neither the immediate level change (β_2_ = 3,168 CNY; *p* = 0.674) nor the post-DRG slope change (β_3_ = −132.93 CNY/month; *p* = 0.061) reached statistical significance.

**Figure 3:**
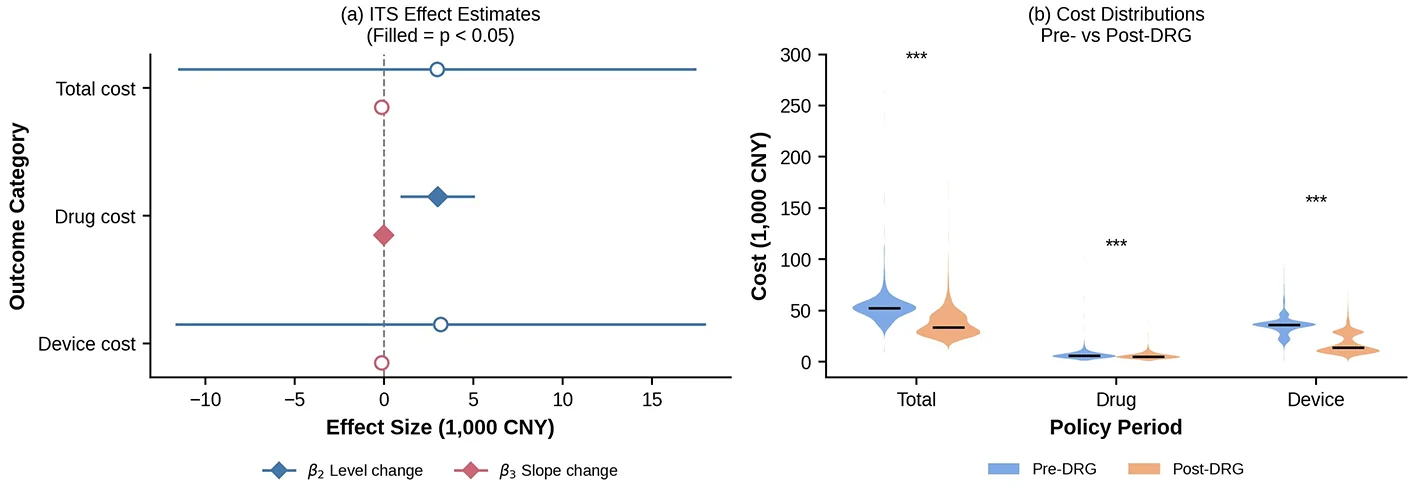
ITS effect estimates and cost distributions. **(a)** Forest plot of immediate level change (β_2_) and monthly slope change (β_3_) from the primary ITS model (CNY × 10^3^). Filled diamonds, *p* < 0.05; open circles, *p* ≥ 0.05. Horizontal bars, 95% confidence intervals; dashed vertical line, null effect. **(b)** Violin plots comparing the full cost distributions between pre-DRG (blue) and post-DRG (orange) periods; horizontal lines denote medians. ***p < 0.001 (two-sample *t*-test).

Figure 3 presents the effect estimates from the primary ITS model alongside the distributional changes in costs between the two periods. Panel (a) shows the immediate level changes (β_2_) and post-intervention slope changes (β_3_) with 95% confidence intervals, highlighting that only the drug cost parameters reached statistical significance. Panel (b) displays the full cost distributions comparing pre-DRG and post-DRG periods, illustrating the substantial reduction in total and device costs, while drug costs showed more modest changes.

### 3.3 Adverse discharge outcomes

The composite adverse discharge rate was 1.06% pre-DRG (11 events /1,041 admissions) and 0.63% post-DRG (10 events /1,580 admissions; χ^2^ = 0.944; *p* = 0.334).

### 3.4 Sensitivity analyses

Results are summarized in Table 3. CP-I deflation to constant 2015 CNY strengthened the pre-existing downward trend for both total (β_1_ = −164.31 CNY/month; *p* < 0.001) and device costs (β_1_ = −154.42 CNY/month; *p* < 0.001), while DRG-specific parameters remained non-significant for both outcomes—confirming that nominal findings are not materially confounded by background price-level changes. Exclusion of 2020 yielded a statistically significant post-DRG slope change for total cost (β_3_ = −146.92 CNY/month; 95% CI [−291.6, −2.3]; *p* = 0.047), demonstrating that COVID-19–related disruptions to elective surgery in 2020 partially attenuated the estimated DRG slope effect in the primary analysis. This sensitivity finding is treated as supportive of directionality, not as primary evidence. The Prais–Winsten AR(1) model produced directionally consistent coefficients for all outcomes, confirming that residual autocorrelation does not substantively alter the primary conclusions. Across all sensitivity specifications, drug cost retained its characteristic transitional level increase and device cost maintained its strong pre-existing declining trend.

**Table 3:** Sensitivity analysis summary—ITS estimates across model specifications.

Model/sensitivity	β_1_ pre-trend	β_2_ level change	β_3_ slope change	*R* ^2^
Total cost/primary (HAC-OLS)	−89.79^**^	2,983 (ns)	−122.76 (ns)	0.703
Total cost/Excl. 2020 (COVID)	−65.63 (ns)	972 (ns)	−146.92^*^	0.703
Total cost/CPI-deflated	−164.31^***^	−2,556 (ns)	−39.88 (ns)	0.778
Total cost/Prais–Winsten	−119.78 (ns)	5,909 (ns)	−125.17 (ns)	—
Total cost/case-mix-adjusted	−91.76^**^	3,039 (ns)	−120.51 (ns)	0.704
Drug cost/primary (HAC-OLS)	−17.07 (ns)	3,006^**^	−27.80^*^	0.225
Drug cost/Excl. 2020	−2.15 (ns)	2,794^**^	−42.72 (ns)	0.242
Drug cost/CPI-deflated	−25.40^*^	2,026^*^	−16.49 (ns)	0.311
Drug cost/case-mix-adjusted	−16.00 (ns)	3,009^**^	−28.80^*^	0.238
Device cost/primary (HAC-OLS)	−104.81^**^	3,168 (ns)	−132.93 (ns)	0.796
Device cost/Excl. 2020	−132.43^***^	−301 (ns)	−105.30 (ns)	0.792
Device cost/CPI-deflated	−154.42^***^	−1,049 (ns)	−64.66 (ns)	0.829
Device cost/case-mix-adjusted	−109.26^**^	3,244 (ns)	−128.19 (ns)	0.801

ns, *p* ≥ 0.05;

^*^p < 0.05;

^**^p < 0.01;

^***^p < 0.001.

Device cost, orthopedic implantable device cost. CPI, consumer price index. *R*^2^ not reported for Prais–Winsten (not directly comparable with OLS).

## 4 Discussion

This eleven-year longitudinal study provides quasi-experimental evidence that DRG-based bundled payment for THA was associated with sustained sub-counterfactual hospitalization costs and a clearly DRG-attributable bimodal drug cost trajectory, without any statistically detectable deterioration in in-hospital adverse discharge outcomes within the limitations of this endpoint.

An important interpretive note is necessary before interpreting the ITS coefficients. The large unadjusted cost reduction alongside a non-significant immediate level change does not indicate a contradiction. Rather, the segmented regression attributes the bulk of the between-period mean difference to the already strongly negative pre-DRG secular trend, while the positive-valued, non-significant β_2_ is a mathematical property of the regression cut-point on a steeply declining trend, not a genuine cost increase at DRG onset. The unadjusted period means and the ITS coefficients should therefore be interpreted jointly: the former document the magnitude of change between periods, while the latter allocate that change to its antecedent-trend and DRG-specific components.

### 4.1 Device cost decline: procurement policy as the primary driver

The 50.5% reduction in mean orthopedic implantable device cost constitutes the most striking finding in this study, yet is also the least attributable to DRG reform *per se*. The pre-intervention ITS model identified a highly significant monthly device cost decline before January 2021 (β_1_ = −104.81 CNY/month, *p* = 0.001), explaining 79.6% of pre-intervention monthly variance (*R*^2^ = 0.796). This trajectory aligns with China's national VBP programme, which achieved centrally tendered prosthetic component price reductions of 50%−80% through successive tendering rounds from 2019 to 2021—a supply-side mechanism operating independently of any payment-side incentive (3). The non-significant β_2_ and β_3_ for device cost confirm that the DRG implementation date produced no discrete additional reduction beyond this antecedent trend.

This does not foreclose a plausible policy-interaction hypothesis: by making expenditure above the DRG tariff a hospital-borne liability, DRG payment may have created institutional disincentives to offset VBP-compressed implant savings through adoption of non-tendered high-cost adjuncts—thereby sustaining device costs at their post-VBP level rather than allowing rebound inflation. The ITS evidence is consistent with this narrative—device costs remained stably depressed without rebound—but the single-arm design cannot exclude the possibility that VBP alone would have achieved the same outcome (7, 12). This interpretation should be understood as a testable hypothesis rather than a confirmed causal mechanism; a multi-site difference-in-differences design comparing institutions subject to VBP alone vs. VBP plus DRG would be required to test it empirically.

### 4.2 Drug cost dynamics: defensive medicine and the learning curve

The drug cost trajectory—significant immediate level increase (β_2_ = 3,006 CNY, *p* = 0.003) followed by significant sustained decline (β_3_ = −27.80 CNY/month, *p* = 0.045)—represents the most statistically robust and clearly DRG-attributable effect in this analysis. Drug cost exhibited no significant pre-DRG secular trend, making the post-DRG bimodal pattern attributable to payment system change rather than antecedent forces.

The initial level surge is consistent with short-term defensive medicine behavior: confronted with a fixed-payment constraint that suddenly threatened the familiar resource envelope for implant expenditure, clinicians may initially have compensated by expanding pharmaceutical intensity—increasing supplemental analgesic protocols, broadening anticoagulation regimens, or utilizing higher-cost perioperative pharmacological agents—within categories perceived as less immediately subject to DRG tariff scrutiny (9, 16). Such within-episode expenditure displacement has been documented in DRG evaluations elsewhere (9, 16). This phase corresponds behaviorally to the disruption period of institutional rule change, during which providers adopt risk-averse practices before new clinical pathways are optimized.

The subsequent sustained monthly decline (approximately CNY 334/year) reflects progressive optimization of perioperative pharmaceutical pathways as clinical pathway committees adapted prescribing protocols to the DRG tariff structure. This disruption-then-rationalization trajectory argues that a minimum 36–48 months of post-intervention observation is required before evaluating DRG's net effect on pharmaceutical stewardship (7).

### 4.3 Clinical quality, tariff adequacy, and moral hazard mitigation

The absence of a statistically significant increase in adverse discharge outcomes (1.06 vs. 0.63%; *p* = 0.334) is reassuring but requires contextualization against both study power limitations and the specific institutional environment.

Given the low baseline adverse event rate (~1%), the study had limited statistical power to detect small differences between periods; the absence of a significant increase is consistent with no detectable deterioration in this in-hospital endpoint but does not constitute definitive confirmation of quality preservation, nor does it extend to post-discharge outcomes not captured by the available administrative data.

Wenzhou presents a structurally advantaged context. As one of China's highest-income coastal cities, its municipal medical insurance pool is substantially better capitalized than the national median, enabling the local healthcare security bureau to calibrate DRG case weights and base rates for THA with sufficient clinical margin to absorb legitimate procedure costs. That said, the theoretical concern with DRG payment is that fixed reimbursement may induce skimping—systematic reduction in care intensity to contain per-admission costs below the tariff (17). Cross-regional Chinese evidence documents heterogeneous responses: in resource-constrained payer environments, hospitals have reportedly engaged in premature discharge, selective refusal of complex admissions, and diagnostic gaming (8). Where DRG tariffs are adequately calibrated, fixed-payment incentives promote care standardization rather than care reduction (5). Policymakers seeking to replicate these results in less well-capitalized payer environments—including most central and western Chinese provinces and many lower-middle-income countries considering DRG adoption—should anticipate greater moral hazard risks and should prioritize tariff adequacy as a foundational precondition for reform (8, 17).

### 4.4 Case-mix evolution: confounder or unrecognized signal?

**Figure 4:**
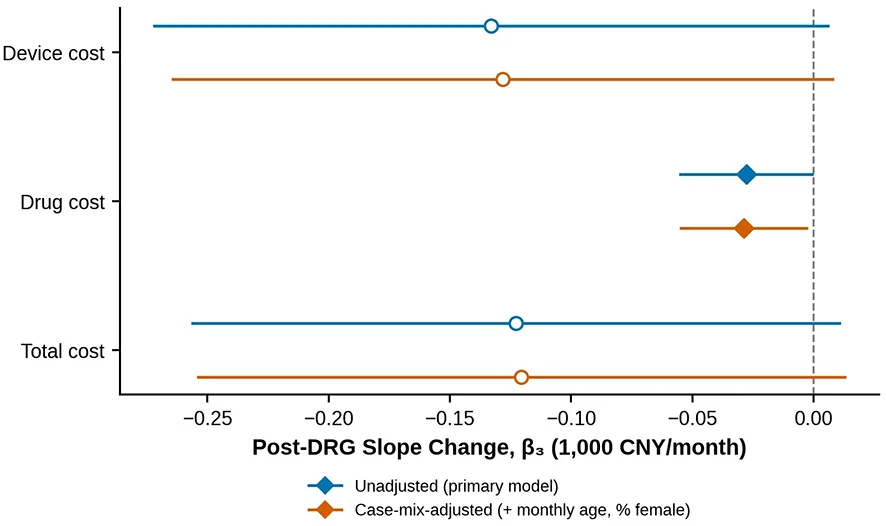
Post-DRG slope change (β_3_) for total, drug, and device cost from the primary (unadjusted) vs. case-mix-adjusted (monthly age and % female added as covariates) ITS models. Filled diamonds, *p* < 0.05; open circles, *p* ≥ 0.05. Coefficients and 95% confidence intervals as reported in Table 3.

The significant increase in mean patient age (65.4–68.9 years; *p* < 0.001) and female proportion (53.8%−65.1%; *p* < 0.001) during the post-DRG period warrants consideration as a potential source of case-mix variation. Two explanations are possible: these changes may reflect the underlying aging trend of the THA-eligible population in China, or they may represent changes in patient composition associated with evolving referral patterns and healthcare delivery under the new payment environment (3, 18). Incorporating monthly mean age and sex distribution into a secondary case-mix-adjusted ITS model did not materially alter the estimated intervention effects (post-DRG slope changes for total, drug, and device costs: −120.51, −28.80, and −128.19 CNY/month in the adjusted model vs. −122.76, −27.80, and −132.93 CNY/month in the primary model) (Figure 4). These findings suggest that the observed cost trajectories were not substantially explained by the measured demographic shifts. Nevertheless, aggregate-level adjustment cannot fully replace patient-level risk adjustment incorporating comorbidity burden, operative indications, and clinical severity; therefore, residual case-mix confounding cannot be completely excluded.

If post-DRG patients were systematically more complex, then the sustained downward cost trajectory represents a genuine resourcefulness achievement: lower costs despite treating a clinically heavier case mix. The case-mix-adjusted sensitivity analysis above is reassuring on this point at the aggregate level, but cannot be fully resolved without patient-level risk adjustment incorporating comorbidity scores (e.g., Charlson Comorbidity Index), surgical indication, and ASA physical status. Future analyses should incorporate case-mix index as a covariate in the ITS model or adopt individual-level regression with demographic adjustment.

### 4.5 International comparisons

The scale of device cost reduction (50.5%) substantially exceeds reductions attributable to DRG adoption alone in Western healthcare systems, where 10%−20% efficiency gains are typical (10). The 50.5% decline reflects China's uniquely powerful VBP–DRG dual-reform environment and should not be extrapolated as a generalizable DRG effect. Researchers in other settings considering DRG adoption should note that equivalent cost reductions require a comparable centralized procurement infrastructure operating in parallel (3). The US Comprehensive Care for Joint Replacement (CJR) model provides the most directly comparable Western bundled-payment evaluation: a mandatory two-year evaluation reported a modest reduction in episode spending without compromising quality (19), a finding echoed by subsequent hospital-level analyses (20) and by spillover analyses in non-mandated markets (21). These single-digit-to-low-teens percentage reductions are an order of magnitude smaller than the device cost decline observed here, underscoring that centralized administrative price-setting, rather than payment reform alone, is likely the dominant mechanism in the Chinese context. European bundled-payment and DRG systems illustrate a further point of contrast: a comparison of DRG classification and reimbursement rules for joint replacement across 11 European countries found substantial heterogeneity in case-mix grouping and only partial ability of DRG variables to explain resource-use variation (22), suggesting that the magnitude and mechanism of any DRG-attributable effect are highly system-specific and cannot be assumed to generalize from one national payment architecture to another. Finally, two recent Chinese interrupted time series evaluations of overlapping procurement-and-payment reforms—centralized volume-based procurement of high-value consumables in Gansu (23) and combined zero-markup and volume-based procurement reform for orthopedic spinal devices (24)—report cost trajectories qualitatively similar to those described here, reinforcing that procurement-side reforms, rather than DRG payment *per se*, are likely the principal driver of implant and consumable cost reduction when the two policies are temporally concurrent.

### 4.6 Strengths and limitations

Strengths include the 11-year observation window (72 pre-intervention and 64 post-intervention monthly observations), Newey–West HAC correction for autocorrelation, deduplication of the analytic cohort prior to analysis, and four pre-specified sensitivity analyses demonstrating directional robustness.

Limitations include the single-center design restricting generalizability. Durbin–Watson statistics below 1.5 in the total and device cost models indicate residual positive autocorrelation even after HAC correction; the Prais–Winsten sensitivity confirms directional robustness, but ARIMA modeling may better capture temporal dynamics in future work. The aggregate monthly ITS model lacks patient-level risk adjustment; the significant post-DRG demographic shift constitutes potential residual case-mix confounding. The composite adverse discharge endpoint does not capture 30-day readmissions, surgical site infection, implant survival, or patient-reported outcomes; registry-linked follow-up studies are required for comprehensive quality assessment. Finally, the temporal overlap of DRG and VBP reforms precludes definitive causal attribution of cost changes to DRG payment alone (12).

## 5 Conclusions

Following DRG implementation, total hospitalization and orthopedic implantable device costs continued their pre-existing downward trajectories, with observed expenditure sustained well below the pre-intervention counterfactual across 64 post-intervention monthly observations. The primary ITS analysis did not identify a statistically significant DRG-specific acceleration of this decline for total or device costs, a finding that predominantly reflects the dominant contribution of antecedent volume-based procurement reform. Drug cost exhibited the most clearly DRG-attributable effect—a significant transitional surge followed by a statistically significant sustained monthly decline consistent with a provider learning-curve response to fixed-payment incentives. No evidence of in-hospital deterioration in adverse discharge outcomes was detected; this underpowered endpoint did not capture post-discharge complications such as 30-day readmission or surgical site infection. Sensitivity analysis excluding the COVID-19–affected year 2020 yielded a statistically significant post-DRG slope change for total cost, reinforcing the directional signal in the primary model.

Collectively, the ITS evidence supports the continued expansion of DRG reform for implant-intensive elective procedures in China, while highlighting two critical preconditions for safe and effective implementation: adequate DRG tariff calibration relative to actual case costs, to prevent quality-cost trade-offs; and explicit accounting for concurrent procurement policy when attributing cost changes to payment reform. Multicentre studies incorporating patient-level risk adjustment, case-mix index covariates, and long-term clinical outcome data are needed to fully characterize the value implications of DRG reform across the full spectrum of Chinese payer environments.

## Data Availability

The raw data supporting the conclusions of this article will be made available by the authors, without undue reservation.
