# Correction: Impact of diagnosis-related group–based bundled payment on hospitalization costs and clinical outcomes in total hip arthroplasty: an interrupted time series analysis from Wenzhou, China

**DOI:** 10.3389/fpubh.2026.1986707

**Published:** 2026-09-07

**Authors:** Frontiers Production Office

**Affiliations:** Frontiers Media SA, Lausanne, Switzerland

**Keywords:** bundled payment, China, cost containment, diagnosis-related groups, health payment reform, interrupted time series, orthopedic implant procurement, total hip arthroplasty

Reviewer's Masoomeh Gholami affiliation should be “Goletan University of Medical Sciences, Iran”.

The original version of this article has been updated.

